# The PedsQL™ Present Functioning Visual Analogue Scales: preliminary reliability and validity

**DOI:** 10.1186/1477-7525-4-75

**Published:** 2006-10-04

**Authors:** Sandra A Sherman, Sarajane Eisen, Tasha M Burwinkle, James W Varni

**Affiliations:** 1 SDSU/UCSD Joint Doctoral Program in Clinical Psychology, 6363 Alvarado Court, #103, San Diego State, San Diego, CA 92120, USA; 2 The Interior Design Program, Department of Consumer Affairs, College of Human Sciences, 308 Spidle Hall, Auburn University, AL 36849, USA; 3 The Children's Hospital at Scott & White, Department of Pediatrics, Texas A&M University Health Science Center, 2401 South 31^st ^Street, Temple, TX 76508, USA; 4 Department of Pediatrics, College of Medicine, Department of Landscape Architecture and Urban Planning, College of Architecture, Texas A&M University, 3137 TAMU, College Station, TX 77843-3137, USA

## Abstract

**Background:**

The PedsQL™ Present Functioning Visual Analogue Scales (PedsQL™ VAS) were designed as an ecological momentary assessment (EMA) instrument to rapidly measure present or at-the-moment functioning in children and adolescents. The PedsQL™ VAS assess child self-report and parent-proxy report of anxiety, sadness, anger, worry, fatigue, and pain utilizing six developmentally appropriate visual analogue scales based on the well-established Varni/Thompson Pediatric Pain Questionnaire (PPQ) Pain Intensity VAS format.

**Methods:**

The six-item PedsQL™ VAS was administered to 70 pediatric patients ages 5–17 and their parents upon admittance to the hospital environment (Time 1: T1) and again two hours later (Time 2: T2). It was hypothesized that the PedsQL™ VAS Emotional Distress Summary Score (anxiety, sadness, anger, worry) and the fatigue VAS would demonstrate moderate to large effect size correlations with the PPQ Pain Intensity VAS, and that patient" parent concordance would increase over time.

**Results:**

Test-retest reliability was demonstrated from T1 to T2 in the large effect size range. Internal consistency reliability was demonstrated for the PedsQL™ VAS Total Symptom Score (patient self-report: T1 alpha = .72, T2 alpha = .80; parent proxy-report: T1 alpha = .80, T2 alpha = .84) and Emotional Distress Summary Score (patient self-report: T1 alpha = .74, T2 alpha = .73; parent proxy-report: T1 alpha = .76, T2 alpha = .81). As hypothesized, the Emotional Distress Summary Score and Fatigue VAS were significantly correlated with the PPQ Pain VAS in the medium to large effect size range, and patient and parent concordance increased from T1 to T2.

**Conclusion:**

The results demonstrate preliminary test-retest and internal consistency reliability and construct validity of the PedsQL™ Present Functioning VAS instrument for both pediatric patient self-report and parent proxy-report. Further field testing is required to extend these initial findings to other ecologically relevant pediatric environments.

## Background

Patient-reported outcomes (PROs) are self-report instruments that directly measure the patient's perceptions of the impact of disease and treatment as clinical trial endpoints, and include multi-item health-related quality of life (HRQOL) instruments, as well as single-item measures (e.g., pain visual analogue scale), daily diaries, treatment adherence, and healthcare satisfaction [1,2]. Pediatric PROs must be sensitive to cognitive development and should include both child self-report and parent proxy-report to reflect their potentially unique perspectives. However, imperfect agreement between self and proxy report, termed cross-informant variance [3], has been consistently documented in the PRO measurement of children with chronic health conditions and healthy children [4]. The demonstration of cross-informant variance and the general acceptance that HRQOL and symptoms derive from an individual's perceptions [5], indicates an essential need in pediatric symptom measurement for reliable and valid pediatric patient self-report instruments for the broadest age range possible. With this in mind, the Pediatric Quality of Life Inventory™ (PedsQL™) was conceptualized as an age-appropriate PRO for a wide age range of children [6,7].

The PedsQL™ Measurement Model was designed as a modular approach to measuring pediatric PROs, developed to integrate the relative merits of generic and disease-specific approaches [6]. Although other pediatric HRQOL instruments exist, including generic measures and disease-specific measures [8,9], it has been an explicit goal of the PedsQL™ Measurement Model [6] to develop and test brief measures for the broadest age group empirically feasible, specifically including child self-report for the youngest children possible. This goal was originally articulated in empirical efforts in the 1980's to measure pain perception in pediatric patients through the development and testing of the Varni/Thompson Pediatric Pain Questionnaire™ (PPQ) for children as young as 5 years of age [10].

The PedsQL™ includes child self-report for ages 5–18 and parent proxy-report for ages 2–18 [7,11]. The items chosen for inclusion were initially derived from the measurement properties of the child self-report scales, while the parent proxy-report scales were constructed to directly parallel the child self-report items. Thus, the development and testing of the PedsQL™ as a pediatric PRO explicitly emphasizes the child's perceptions. Given that the PedsQL™ Measurement Model integrates generic core scales and disease-specific symptom scales into one measurement system, and that the origins of the PedsQL™ derived from the initial conceptual model of the PPQ [10,12-14], then a logical extension of the PedsQL™ Measurement Model entailed the integration of at-the-moment or present functioning visual analogue scales (VASs) based on the PPQ's well-established age-appropriate VAS format.

Developments in ecological momentary assessment (EMA) suggest the benefits of measuring symptoms at-the-moment in ecologically relevant environments [15]. The measurement of present or at-the-moment functioning has been well established for pediatric pain intensity using VASs for over 20 years [16,17]. As reviewed by McGrath [18], the VAS, although deceptively simple, has demonstrated the measurement properties necessary for a pain symptom measure in numerous experimental and clinical pain studies. Historically, the VAS had been used extensively with adult pain patients because of its sensitivity and reproducibility [19], and to a lesser extent in adults patients with mood and anxiety disorders [20]. More recently, the VAS has been utilized as a single-item unidimensional measure of fatigue [21] and global HRQOL in adult patients [22].

As a continuous measurement scale, the VAS potentially avoids the clustering of symptom reports that may occur with single-item categorical response methods [23]. In both children and adults, the pain VAS has demonstrated excellent construct validity in postoperative medication studies, showing the expected reduction in pain subsequent to analgesia intake [23-26], and in pediatric chronic musculoskeletal pain, demonstrating the expected increase in perceived pain intensity with greater rheumatic disease activity [10].

A 10 cm (100 mm) line length has been found to have the smallest measurement error in comparison to line lengths of 5 and 20 cm [27]. In adults, vertical or horizontal VASs exhibit extremely high correlation (*r *= 0.99) [28]. From a psychophysical measurement perspective, the VAS is considered a direct scaling method and a form of cross-modality matching in which the length of the line is adjusted to match the intensity of the pain [18,19]. Price et al. have investigated the measurement properties of the VAS in a series of psychophysical studies, demonstrating that the VAS has ratio rather than interval scale properties [29]. Thus, in addition to properties of interval scales which reflect equal distances in the variables being quantitatively ordered, the ratio scale indicates a true zero point [30].

The VAS has been shown to be a reliable and valid PRO measure of pediatric patients' pain intensity in children as young as 5 years of age [10,31,32]. Using the PPQ Pain Intensity VAS format as a template [10], we subsequently developed the PedsQL™ Present Functioning Visual Analogue Scales (PedsQL™ VAS) to measure anxiety, sadness, anger, worry, fatigue, and pain as experienced at-the-moment. Previously, in a pilot study which was part of a larger investigation of a healing garden adjacent to a pediatric cancer center, the PedsQL™ VAS demonstrated the hypothesized findings that self-reported anxiety, sadness, anger, worry, fatigue, and pain levels would be lower in individuals when in the healing garden environment than in individuals when in the hospital inpatient and clinic environment [33]. Although these data suggest the initial construct validity of the PedsQL™ VAS, the sample was small and included a mixed sample of pediatric patients, parents, and staff reports. Thus, as part of a larger architecture doctoral dissertation study investigating the effects of the hospital environment on children, the PedsQL™ VAS were administered to pediatric patients and their parents upon admission to their hospital room and 2 hours subsequently.

The experience of hospitalization entails many of the "precipitants" (disease, injury, stress, medical procedures) of pain and other symptoms posited by the Biobehavioral Model of Pediatric Pain [34,35], and as such, provides an ecologically relevant context in which to test the preliminary reliability and validity of the PedsQL™ symptom VASs. Hospitalized children may find the experience stressful [36], with elevated stress responses associated with severity of illness or injury and number of invasive procedures [37,38]. In response to these potential stressors, hospitals have developed child life programming, including pre-hospitalization preparation and subsequent support immediately at the time of hospitalization and during the hospitalization period, all intended to ameliorate the purported negative psychological reactions which may occur during hospitalization and the associated medical procedures [37,39].

One of the major positive changes in the typical pediatric hospitalization experience has been in the routine implementation of a policy of open visitation rights for parents, including overnight stays, which has resulted in parental social support and coping guidance throughout the hospitalization experience, resulting in a significant attenuation of many of the emotional distress symptoms previously documented in the older pediatric hospitalization literature [40]. However, it might be expected that children's reactions to the hospital experience will vary substantially across patients, resulting in a mean reduction of symptoms at the group level as a result of contemporary visitation policies, but probable large individual coping differences among patients based on such factors as age, injury or disease characteristics, and the number and types of medical procedures administered [40].

Based on the extant pediatric hospitalization stress and coping literature [40], we thus hypothesized pediatric patients' emotional distress ratings would be in the lower range of severity at the group level, but given individual differences in coping abilities and parental support, we hypothesized considerable variability in patient and parent VAS ratings. Further, based on the previous literature with pediatric and adult patients, we anticipated medium to large effect size correlations between emotional distress and fatigue with pain intensity [41-46]. We further hypothesized mean pain VAS scores in the mild range since the assessments were conducted prior to medical procedures, but anticipated considerable variability given differing disease and injury characteristics. Finally, we expected that distress ratings would decrease over the 2-hour interval as patients accommodated to their hospital rooms, and that parent-child concordance on the VASs would increase with time given parents' prototypical constant presence and vigilance during the initial hospitalization period [40]. Finally, we expected that test-retest reliability would be attenuated during the 2-hour retest interval in part due to this anticipated decrease in symptoms over time.

## Method

### Participants and settings

As part of a larger architecture doctoral dissertation study on the effects of the hospital environment on pediatric patients, all children that were admitted to the hospital in the 5–17 age group and their parents during the recruitment period were asked to participate. Seventy-five families were approached, and 70 families agreed to participate (93% participation rate). The reasons for declining participation included that the child was too emotionally upset or in pain (secondary to sickle cell anemia crisis) to participate.

Seventy children and their parents completed the PedsQL™ Present Functioning Visual Analogue Scales. The children were ages 5–17 (Mean age = 10.61, S.D. = 3.54; 40 boys, 30 girls), No ethnicity data were collected. The children were hospitalized for treatment of acute (e.g., infections, accidents, injuries) and chronic (e.g., cystic fibrosis respiratory treatment, sickle cell anemia) conditions as reported by the inpatient nursing staff. However, the larger architecture doctoral dissertation study of which these data are derived from did not have access to individual patient medical records, and thus it was not possible to link patient characteristics regarding medical condition to the VAS data. Pediatric patients and their parents were approached for permission to be part of the study once they had been admitted into their hospital room.

Pediatric patients and their parents completed the PedsQL™ VAS upon admittance, after they had consented to be part of the study, and then two hours later, before any medical procedures were performed. Data were collected concurrently at two sites: Scott & White Memorial Hospital in Temple, Texas, and Christus St. Elizabeth's Hospital in Beaumont, Texas. The length of recruitment was 3 weeks for the Scott & White Memorial Hospital and 6 weeks for the Christus St. Elizabeth's Hospital. Institutional Review Board approval was obtained from both of these institutions prior to data collection.

### PedsQL™ Present Functioning Visual Analogue Scales

The PedsQL™ VAS instrument contains six items that measure anxiety, sadness, anger, worry, fatigue, and pain at the present moment, and where derived from the PedsQL™ Emotional Functioning Scale items [7] and PedsQL™ Fatigue Scale items [43], as well as the Varni/Thompson PPQ Pain Intensity VAS [10]. The PedsQL™ VAS utilizes 100 mm lines anchored at one end with a happy face and at the other end with a sad face based on the original PPQ VAS format. The instructions ask participants to "Please put a mark on each line that best shows how you feel now. If you have no problem and feel fine, put a mark at the end of the line by the happy face. If you have some problems and do not feel that well, put a mark near the middle of the line. If you feel very bad or have lots of problems, put a mark by the sad face." The PedsQL™ VAS Total Symptom Score is calculated by taking the average of all six items, while the Emotional Distress Summary Score represents the mean of the anxiety, sadness, anger, and worry items, similar to the PedsQL™ Emotional Functioning Scale [7]. Pain and fatigue were conceptualized as individual symptom items, and where included in the Total Symptom Score.

### Varni/Thompson Pediatric Pain Questionnaire VAS

The Varni/Thompson Pediatric Pain Questionnaire (PPQ) Pain Intensity VAS measurement properties were initially tested in pediatric rheumatology patients as young as 5 years old and their parents, demonstrating greater perceived pain intensity associated with greater rheumatic disease activity [10]. Subsequent research studies have supported the measurement properties of the PPQ Pain Intensity VAS internationally [12,42,47-56], including responsiveness to treatment effects [57,58].

### Statistical analysis

Test-retest reliability was evaluated by computing both Pearson Product Moment Correlation coefficients and Intraclass Correlations (ICC) for each item and scale from Time 1 to Time 2. Pearson Product Moment Correlation coefficient effect sizes are designated as small (.10–.29), medium (.30–.49), and large (≥.50) [59]. Intraclass correlations (ICC) are designated as ≤ 0.40 poor to fair agreement, 0.41–0.60 moderate agreement, 0.61–0.80 good agreement, and 0.81–1.00 excellent agreement [60,61]. Scale internal consistency reliability was determined by calculating Cronbach's coefficient alpha [62]. Scales with reliabilities of 0.70 or greater are recommended for comparing patient groups [63].

Construct validity for the PedsQL™ VAS was investigated through an analysis of the intercorrelations among the emotional distress and fatigue items with the benchmark PPQ Pain Intensity VAS criterion. Computing the intercorrelations among scales provides initial information on the construct validity of an instrument [30]. Paired sample t-tests were conducted to compare scores from Time I to Time 2.

The concordance between patient self-report and parent proxy-report was determined through computing both Pearson Product Moment Correlation coefficients and Intraclass Correlations (ICC) at both Time 1 and Time 2. Given no significant differences between sites in terms of VAS scores after a Bonferroni correction for the number of tests conducted, data were pooled across sites. Statistical analyses were conducted with SPSS.

## Results

### Descriptives

Means and standard deviations for pediatric patient self-report and parent proxy-report across the different age groups are presented in Table 1 for Time 1 and Time 2. There was considerable variability at the item and scale level, with the standard deviations exceeding the mean for a number of the individual VAS scores, suggesting considerable individual differences.

**Table 1:** PedsQL™ Present Functioning Visual Analogue Scales Descriptives (N = 70)

	**Ages 5–7 (n = 18)**				**Ages 8–12 (n = 31)**				**Ages 13–17 (n = 21)**			
**PedsQL™ Items**	Patient Self-Report		Parent Proxy-Report		Patient Self-Report		Parent Proxy-Report		Patient Self-Report		Parent Proxy-Report	

	**Mean**	**SD**	**Mean**	**SD**	**Mean**	**SD**	**Mean**	**SD**	**Mean**	**SD**	**Mean**	**SD**

**Time 1 Scores**												
Total Score	25.60	19.04	29.92	18.28	28.17	20.70	38.88	22.10	28.87	19.54	35.99	20.89
Emotional Distress Summary Score	21.60	23.82	23.76	16.90	19.31	19.48	33.77	21.73	20.86	19.67	31.37	21.55
Anxiety VAS	25.00	36.10	23.61	26.83	17.26	22.94	30.48	24.54	25.57	28.02	30.71	29.25
Sadness VAS	22.78	35.45	17.83	24.04	17.58	24.22	38.39	34.67	15.00	17.39	30.48	27.15
Anger VAS	2.22	4.92	7.22	11.40	9.42	16.59	15.23	20.22	6.90	13.82	20.95	24.52
Worry VAS	36.39	41.33	46.39	26.33	32.97	36.52	50.97	29.62	35.95	34.77	43.33	32.07
Fatigue VAS	38.89	41.50	39.17	35.16	54.19	35.59	55.32	33.96	46.90	35.93	44.52	32.94
Pain VAS	28.33	34.13	45.28	34.75	37.58	35.91	42.90	34.27	42.86	30.72	45.95	31.37
**Time 2 Scores**												
Total Score	20.65	22.21	20.62	23.78	23.02	21.58	31.48	21.81	24.56	14.68	29.29	16.15
Emotional Distress Summary Score	17.43	21.02	17.32	22.91	17.84	20.34	25.60	22.73	17.38	16.77	23.10	15.51
Anxiety VAS	13.89	26.04	18.06	28.03	15.78	24.59	23.71	25.92	17.62	22.67	23.47	23.46
Sadness VAS	22.78	33.96	20.83	33.62	14.68	25.91	22.10	28.04	15.00	18.44	19.52	17.60
Anger VAS	3.06	3.89	6.11	10.23	10.32	21.68	20.48	28.59	5.48	6.87	15.71	22.21
Worry VAS	30.00	39.78	24.28	28.03	30.48	34.98	36.13	29.12	31.43	28.95	33.57	27.21
Fatigue VAS	27.78	35.45	26.94	32.36	35.16	32.42	47.58	36.67	42.62	28.18	43.57	25.65
Pain VAS	26.39	37.57	27.50	37.54	31.61	32.54	38.87	32.63	35.24	25.37	39.76	23.21

Note: 0–100 VAS scores; higher values = higher symptom levels.

### Test-retest reliability

Test-retest reliability from Time 1 to Time 2 demonstrated mainly moderate to larger effect sizes for patient self-report and parent proxy-report (Table 2).

**Table 2:** PedsQL™ Present Functioning Visual Analogue Scales Test-Retest Reliability.

**Item**	**Patient Report**	**Parent Report**
**Total Score**	.73** .85	.65** .79
**Emotional Distress Summary Score**	.65** .78	.61** .76
**Anxiety**	.53** .69	.52** .69
**Sadness**	.53** .70	.55** .70
**Anger**	.36** .52	.74** .84
**Worry**	.64** .78	.45** .62
**Fatigue**	.59** .74	.53** .69
**Pain**	.60** .75	.73** .84

Patient Self-Report and Parent Proxy-Report Time 1 to Time 2 Correlations.

*p < .05, **p < .01 (2-tailed).

Note: Effect sizes are designated as small (.10–.29), medium (.30–.49), and large (≥.50) for Pearson Product Moment Correlations. Intraclass Correlations (ICCs) are designated as ≤ 0.40 poor to fair agreement, 0.41–0.60 moderate agreement, 0.61–0.80 good agreement, and 0.81–1.00 excellent agreement. Intraclass Correlation Coefficients are listed in bold below Pearson Product Moment Correlations.

### Internal consistency reliability

Internal consistency reliability alpha coefficients for the PedsQL™ VAS are presented in Table 3. The Total Symptom Score and Emotional Distress Summary Score exceeded the minimum reliability standard for group comparison of 0.70 for the majority of patient self-report and parent proxy-report scales.

**Table 3:** PedsQL™ Present Functioning Scales Internal Consistency Reliability for Patient Self-Report and Parent Proxy-Report

	**Ages 5–7 (n = 18)**	**Ages 8–12 (n = 31)**	**Ages 13–17 (n = 21)**	**Total N = 70**
***Patient Report***				
**Time 1**				
Total Score	0.54	0.79	0.79	**0.72**
Emotional Distress Summary Score	0.70	0.74	0.80	**0.74**
**Time 2**				
Total Score	0.79	0.84	0.71	**0.80**
Emotional Distress Summary Score	0.69	0.74	0.82	**0.73**
***Parent Report***				
**Time 1**				
Total Score	0.80	0.83	0.80	**0.80**
Emotional Distress Summary Score	0.71	0.79	0.76	**0.76**
**Time 2**				
Total Score	0.84	0.81	0.78	**0.84**
Emotional Distress Summary Score	0.89	0.83	0.61	**0.81**

### Construct validity

Tables 4 and 5 present the correlations between PedsQL™ Present Functioning VAS items and scale scores for patient self-report and parent proxy-report at Time 1 and Time 2. The emotional distress and fatigue VAS ratings demonstrated mostly significant correlations in the medium to large effect size range with the pain VAS criterion.

**Table 4:** PedsQL™ Present Functioning Visual Analogue Scales Time 1 Correlations: Patient Self-Report Above the Diagonal, Parent Proxy-Report Below the Diagonal, Patient/Parent Correlations on the Diagonal.”

	**Total**	**Emotional**	**Anxiety**	**Sadness**	**Anger**	**Worry**	**Fatigue**	**Pain**
**Total**	.45**	.89**	.68**	.71**	.48**	.78**	.55**	.74**
**Score**	**.62**	*.94*	*.78*	*.81*	*.62*	*.79*	*.62*	*.78*
**Emotional**	.93**	.40**	.81**	.77**	.56**	.85**	.19**	.48**
**Distress Summary Score**	*.96*	**.57**	*.87*	*.86*	*.69*	*.84*	*.27*	*.60*
**Anxiety**	.75**	.81**	.34**	.51**	.34**	.56**	.04	.35**
**VAS**	*.84*	*.88*	**.50**	*.67*	*.43*	*.70*	*.08*	*.51*
**Sadness**	.75**	.79**	.47**	.41**	.38**	.48**	.12	.48**
**VAS**	*.82*	*.85*	*.63*	**.58**	*.48*	*.62*	*.20*	*.63*
**Anger**	.56**	.65**	.40**	.44**	.19	.35**	.09	.23
**VAS**	*.72*	*.78*	*.55*	*.58*	**.31**	*.37*	*.11*	*.27*
**Worry**	.77**	.81**	.63**	.46**	.31**	.38**	.26*	.39**
**VAS**	*.84*	*.86*	*.77*	*.63*	*.45*	**.54**	*.42*	*.56*
**Fatigue**	.67**	.43**	.30*	.38**	.11	.46**	.19	.37**
**VAS**	*.75*	*.55*	*.45*	*.55*	*.18*	*.62*	**.32**	*.53*
**Pain**	.76**	.57**	.49**	.47**	.40**	.41**	.44**	.38**
**VAS**	*.82*	*.68*	*.64*	*.64*	*.53*	*.57*	*.61*	**.55**

*p < .05, **p < .01 (2-tailed).

Note: Effect sizes are designated as small (.10–.29), medium (.30–.49), and large (≥.50) for Pearson Product Moment Correlations. Intraclass Correlations (ICCs) are designated as ≤ 0.40 poor to fair agreement, 0.41–0.60 moderate agreement, 0.61–0.80 good agreement, and 0.81–1.00 excellent agreement. Intraclass Correlation Coefficients are listed in bold below Pearson Product Moment Correlations.

**Table 5:** PedsQL™ Present Functioning Visual Analogue Scales Time 2 Correlations: Patient Self-Report Above the Diagonal, Parent Proxy-Report Below the Diagonal, Patient/Parent Correlations on the Diagonal

	**Total**	**Emotional**	**Anxiety**	**Sadness**	**Anger**	**Worry**	**Fatigue**	**Pain**
**Total**	.66**	.92**	.70**	.75**	.40**	.82**	.69**	.81**
**Score**	**.80**	*.96*	*.81*	*.84*	*.55*	*.83*	*.76*	*.84*
**Emotional**	.93**	.58**	.77**	.83**	.44**	.88**	.40**	.58**
**Distress Summary Score**	*.97*	**.73**	*.86*	*.88*	*.60*	*.86*	*.52*	*.68*
**Anxiety**	.79**	.86**	.53**	.49**	.13	.59**	.27*	.46**
**VAS**	*.87*	*.92*	**.69**	*.66*	*.21*	*.72*	*.41*	*.61*
**Sadness**	.80**	.81**	.63**	.43**	.30*	.62**	.25*	.54**
**VAS**	*.87*	*.88*	*.77*	**.60**	*.41*	*.75*	*.39*	*.69*
**Anger**	.59**	.66**	.37**	.41**	.56**	.22	.16	.25*
**VAS**	*.74*	*.79*	*.53*	*.58*	**.67**	*.28*	*.21*	*.33*
**Worry**	.80**	.85**	.75**	.55**	.39**	.60**	.45**	.47**
**VAS**	*.87*	*.90*	*.86*	*.71*	*.56*	**.74**	*.62*	*.63*
**Fatigue**	.75**	.53**	.46**	.49**	.21	.52**	.60**	.60**
**VAS**	*.81*	*.65*	*.61*	*.64*	*.33*	*.68*	**.75**	*.75*
**Pain**	.74**	.53**	.38**	.54**	.38**	.39**	.54**	.60**
**VAS**	*.81*	*.65*	*.54*	*.69*	*.54*	*.56*	.70	**.75**

. *p < .05, **p < .01 (2-tailed).

Note: Effect sizes are designated as small (.10–.29), medium (.30–.49), and large (≥.50) for Pearson Product Moment Correlations. Intraclass Correlations (ICCs) are designated as ≤ 0.40 poor to fair agreement, 0.41–0.60 moderate agreement, 0.61–0.80 good agreement, and 0.81–1.00 excellent agreement. Intraclass Correlation Coefficients are listed in bold below Pearson Product Moment Correlation.

Table 6 demonstrates a general trend toward lower scores over time for patient self-report and significantly lower scores for parent proxy-report. The Total Symptom Score was significantly lower at Time 2 for patient self-report, with the fatigue VAS the only significantly lower score at Time 2. Most items and scales for parent proxy-report were significantly lower at Time 2 except for the anger VAS, which showed a nonsignficant increase at Time 2.

**Table 6:** PedsQL™ Present Functioning Visual Analogue Scores: Time 1 to Time 2 Differences

		**Time 1**		**Time 2**				
**Item**	**N**	**Mean**	**SD**	**Mean**	**SD**	**Difference**	**Effect Size**	**t**
**Child Report**								
Total	70	27.72	19.70	22.87	19.72	-4.85	.25	2.81**
Emotional Distress Summary Score	70	20.36	20.43	17.60	19.25	-2.76	.13	NS
Anxiety	70	21.74	28.17	15.89	24.10	-5.85	.21	NS
Sadness	70	18.14	25.71	16.86	26.21	-1.28	.05	NS
Anger	70	6.81	13.76	7.00	15.21	+0.19	.01	NS
Worry	70	34.74	36.79	30.64	34.13	-4.10	.11	NS
Fatigue	70	48.07	37.26	35.50	32.06	-12.57	.33	3.31***
Pain	70	36.79	33.93	31.36	31.71	-5.43	.15	NS
**Parent Report**								
Total	70	35.71	20.85	28.03	21.02	-7.68	.37	3.69***
Emotional Distress Summary Score	70	30.48	20.67	22.72	20.85	-7.76	.38	3.53***
Anxiety	70	28.79	26.39	22.21	25.52	-6.58	.25	2.17*
Sadness	70	30.73	30.80	21.00	26.67	-9.73	.32	2.95**
Anger	70	14.89	20.27	15.36	23.63	+0.47	.02	NS
Worry	70	47.50	29.35	32.31	28.30	-15.19	.52	4.18***
Fatigue	70	47.93	34.17	41.07	33.27	-6.86	.20	NS
Pain	70	44.43	33.10	36.21	31.51	-8.22	.25	2.88**

*p < .05, **p < .01, ***p < .001.

Note: Effect sizes are designated as small (.20), medium (.50), and large (.80).

### Parent/Child concordance

The parent/child concordance intercorrelations matrix is shown in Tables 4 and 5 for Time 1 and Time 2, with Pearson correlations and ICCs in the small to medium range at Time 1, and in the large range at Time 2.

## Discussion

This study presents the preliminary test-retest and internal consistency reliability and construct validity of the PedsQL™ Present Functioning Visual Analogue Scales for anxiety, sadness, anger, worry, fatigue and pain. Most test-retest reliabilities were in the large effect size range. The majority of internal consistency reliabilities met or exceeded the recommended minimum alpha coefficient standard of 0.70 for group comparisons. Preliminary construct validity of the PedsQL™ Present Functioning VAS was demonstrated through the intercorrelations among the emotional distress and fatigue VAS rating with the well-established PPQ VAS pain intensity criterion.

These findings have certain limitations. The data presented here were collected in the context of a larger architecture doctoral dissertation study investigating the effects of the hospital environment on children. As such, the larger study was not specifically designed to test the measurement properties or establish the validity of the PedsQL™ Present Functioning VAS. For optimal testing of a present functioning scale, it would be useful to test for subject characteristics that might predict VAS scores, such as injury severity and chronic health condition. Since these data were not collected, it was not possible to separate the patients into known groups in order to determine whether the VAS was able to differentiate patients based on established diagnostic criteria. The 2-hour interval was selected to minimize the probability of the occurrence of medical procedures which would have substantially affected the test-retest reliability calculations. However, the short test-retest interval may have potentially inflated the test-retest reliability and attenuated possible changes in scores from Time 1 to Time 2. Further, given that previous research with the PPQ Pain Intensity VAS has demonstrated a relationship to children's coping strategies [49], and research with adults has demonstrated a relationship between daily recording of pain, negative mood, and perceived support [64], then future research should also study the potential covariates of at-the-moment functioning in pediatric patients that would inform the validation process.

As ecological momentary assessment (EMA) research with adult patients demonstrates the utility of at-the-moment assessment in disentangling the interrelationships between such diverse constructs as pain, mood, fatigue, coping, and social support through a daily process analysis, the application of these methods and technologies to the pediatric population will requirement measurement instruments that are developmentally appropriate. The PedsQL™ Present Functioning Visual Analogue Scales represent one approach to meeting that need.

## Conclusion

This study demonstrates preliminary reliability and validity of the PedsQL™ Present Functioning Visual Analogue Scales for both child self-report and parent-proxy report. The PedsQL™ VAS was designed as a pediatric measurement instrument for the ecological momentary assessment of anxiety, sadness, anger, worry, fatigue, and pain in pediatric patients ages 5–18.

## Abbreviations

PedsQL™ Pediatric Quality of Life Inventory™

PRO Patient-Reported Outcomes

HRQOL Health-Related Quality of Life

VAS Visual Analogue Scale

## Competing interests

The author(s) declare that they have no competing interests.

## Authors' contributions

JWV and SAS designed the instrument. SAS and JWV drafted the manuscript. SAS performed the statistical analyses. SJE conceptualized the rationale and design of the original dissertation study from which the data were collected. JWV and TMB consulted on the statistical analyses. TMB participated in manuscript preparation. All authors read and approved the final manuscript.
